# Carbon Microsphere-Supported Metallic Nickel Nanoparticles as Novel Heterogeneous Catalysts and Their Application for the Reduction of Nitrophenol

**DOI:** 10.3390/molecules26185680

**Published:** 2021-09-18

**Authors:** Melinda Krebsz, László Kótai, István E. Sajó, Tamás Váczi, Tibor Pasinszki

**Affiliations:** 1 School of Chemistry, Monash University, Clayton, VIC 3800, Australia; melinda.krebsz@gmail.com; 2 Institute of Materials and Environmental Chemistry, Research Centre of Natural Sciences, Hungarian Academy of Sciences, P.O. Box 286, 1519 Budapest, Hungary; kotai.laszlo@ttk.hu; 3 János Szentágothai Research Centre, University of Pécs, Ifjúság u. 20, 7624 Pécs, Hungary; istvan.sajo@gmail.com; 4 Wigner Research Centre for Physics, Eötvös Loránd Research Network, Konkoly-Thege Miklós út 29-33, 1121 Budapest, Hungary; vaczi.tamas@wigner.hu; 5 Department of Chemistry, School of Pure Sciences, College of Engineering, Science and Technology, Fiji National University, Samabula, Suva P.O. Box 3722, Fiji

**Keywords:** Ni nanoparticles, XRD, SEM, Raman, carbon microsphere, heterogeneous catalysis

## Abstract

Nickel nanoparticles are gaining increasing attention in catalysis due to their versatile catalytic action. A novel, low-cost and facile method was developed in this work to synthesize carbon microsphere-supported metallic nickel nanoparticles (Ni-NP/C) for heterogeneous catalysis. The synthesis was based on carbonizing a polystyrene-based cation exchange resin loaded with nickel ions at temperatures between 500 and 1000 °C. The decomposition of the nickel-organic framework resulted in both Ni-NP and carbon microsphere formation. The phase composition, morphology and surface area of these Ni-NP/C microspheres were characterized by powder X-ray diffraction, Raman spectroscopy, scanning electron microscopy and BET analysis. Elemental nickel was found to be the only metal containing phase; fcc-Ni coexisted with hcp-Ni at carbonization temperatures between 500 and 700 °C, and fcc-Ni was the only metallic phase at 800–1000 °C. Graphitization and carbon nanotube formation were observed at high temperatures. The catalytic activity of Ni-NP/C was tested in the reduction of 4-nitrophenol to 4-aminophenol by sodium borohydride, and Ni-NP/C was proved to be an efficient catalyst in this reaction. The relatively easy and scalable synthetic method, as well as the easy separation and catalytic activity of Ni-NP/C, provide a viable alternative to existing nickel nanocatalysts in future applications.

## 1. Introduction

Metallic nanoparticles (NPs) have small size, involving dimensions of less than 100 nm, various shapes, and high specific surface area; they exhibit markedly different properties than their larger counterparts, such as powders, grains or granules. The large surface to volume ratio of NPs results in enhanced reactivity, and one of the most important fields of their application is heterogeneous catalysis. Catalysts play a key role in chemical transformations and provide countless opportunities for synthesizing compounds in both academia and chemical industry. Currently, for example, more than 90% of all industrial chemicals are produced with the aid of catalysts [1]. Nickel nanoparticles (Ni-NPs) are gaining increasing attention in nanocatalysis due to their versatile catalytic action and potential to replace noble metal catalysts [2,3,4,5,6,7]. Ni-NPs are cheaper and less toxic than their noble metal counterparts [8,9,10]. NPs’ application, however, is hampered due to their self-aggregation and difficulties in their separation from reaction media by filtration. Expensive recovery methods, and also expensive catalyst systems, render the usage of catalytic assembly uneconomical. One of the possible approaches to mitigate challenges due to aggregation and catalyst separation is the deposition of NPs on support materials. Supports not only stabilize NPs, and provide easy catalyst and product separation, but influence the catalytic behavior of the supported NPs by taking place, e.g., in electron transfer processes. To develop innovative synthetic methods for heterogeneous catalysts with enhanced activity, improved stability, excellent reusability and lower production cost is therefore continuously desired. Considering these challenges, our aim in this work was to find a novel route to a Ni-NP catalyst supported on carbon spheres with diameters in the micrometer domain or larger to assist in easy recovery by filtration. Our working hypothesis was that (1) carbonizing an organic polymer in inert atmosphere would lead to a carbon support, and if polymer chains were cross-linked, carbonization might lead to a robust carbon block, rather than to fragile ashes, (2) finely dispersed nickel ions in the polymer matrix would be good sources of Ni NPs after reduction and nickel atom diffusion at high temperatures, (3) carbon support would stabilize Ni NPs and aid the easy separation of catalyst particles by filtration, and (4) surface Ni NPs could act as catalysts in various chemical reactions. To realize this, cation exchange resins seemed to be feasible starting materials, and in this paper, we prove that both Ni NPs and carbon supports can be simultaneously prepared by carbonizing nickel ion-loaded, styrene-divinylbenzene-based cation exchange resins without the aid of any additional reducing agent. In addition, the catalytic activity of synthesized carbon-supported Ni-NPs was tested on a well-known model reaction, namely the reduction of 4-nitrophenol (4NP) to 4-aminophenol (4AP) by NaBH_4_. This latter industrially important reaction is ideal for testing catalytic performance of our nickel NP catalyst, because (1) the reaction does not proceed at room temperature without a catalyst, (2) the sole product of the reaction is 4AP, (3) the reaction follows a pseudo-first-order kinetics in the presence of a large excess of NaBH_4_, and (4) the reaction can be easily monitored visually by following a color change from yellow (4NP) to colorless (4AP) or by UV/vis spectroscopy following the decrease in the absorption band of 4-nitrophenolate anion [11,12,13,14,15,16].

This paper presents a novel method for synthesizing nickel NPs-decorated carbon microspheres (Ni-NP/C), the determination of their phase composition and surface morphology, and a preliminary study of their catalytic performance in 4NP reduction.

## 2. Results and Discussion

### 2.1. Synthesis and Characterization of Ni-NP/C Catalysts

For synthesizing the carbon support and Ni NPs, a commercial styrene-based cation exchanger resin was selected, which contained divinylbenzene crosslinker and acrylonitrile modifier, as well as iminodiacetate (–CH_2_N(CH_2_COOH)_2_) functional groups to bind nickel ions. The resin had a nominal binding capacity of 1 mol nickel ion per 1 dm^3^ resin. The resin was saturated with nickel ions using aqueous nickel sulfate solution (see Materials and Methods section below). The iminodiacetate chelating groups selectively bind one equivalent of Ni(II) ions, and therefore the functional groups of the loaded resin can be described as [–CH_2_N(CH_2_COO^−^)_2_Ni^2+^(H_2_O)_x_], where Ni ions are chelated by the nitrogen and two oxygen atoms of the iminodiacetate groups; the coordination sphere of nickel ions is expected to be saturated by water molecules. The nickel content on the surface of the loaded resin was estimated using the semi-quantitative energy dispersive X-ray analysis (EDX) method, and was found to be 7.1%.

Carbonization of the loaded and dried resin was conducted in nitrogen atmosphere at 500, 600, 700, 800, 900, and 1000 °C for 2, 4, and 8 h to investigate the effect of temperature and thermolysis time. All eighteen samples prepared were black and contained spherical particles, and were attracted by a magnet.

**Figure 1:**
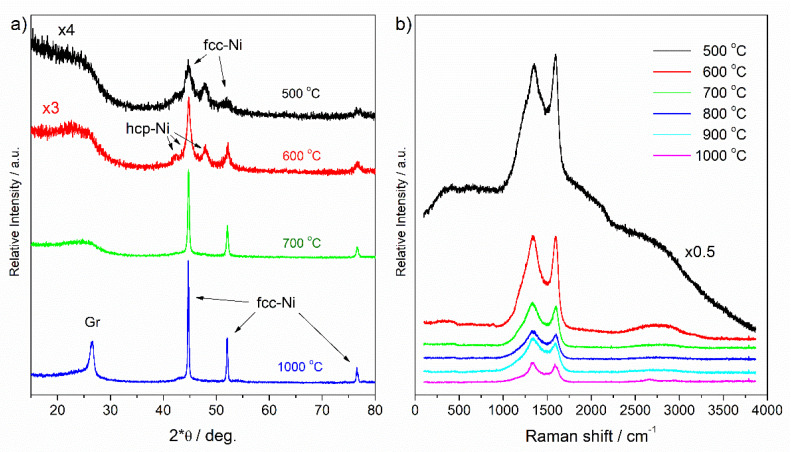
(**a**) Powder X-ray diffractogram of selected Ni-NP/C microspheres carbonized for 8 h (see Table 1), (**b**) Raman spectra of Ni-NP/C microspheres carbonized for 4 h (carbonization temperatures are indicated in Figures).

The phase composition of synthesized materials was determined using powder X-ray diffraction (PXRD) and Raman spectroscopy (Figure 1). Representative PXRD diffractograms are shown in Figure 1a. Only metallic nickel was detected by PXRD as the sole metal containing crystalline phase after carbonization between 500 and 1000 °C (Table 1). The cubic fcc-Ni phase formed together with the hexagonal hcp-Ni at lower carbonization temperatures between 500 and 700 °C, and only fcc-Ni was detected at 800–1000 °C.

The average crystallite sizes of nickel NPs were very small at 500 °C carbonization (about 10 nm), increased gradually with an increasing carbonization temperature, and become larger than 100 nm at 1000 °C. It is interesting to note that the carbon matrix stabilized the meta-stable hcp-Ni at lower temperatures. hcp-Ni, however, gradually recrystallized to the stable fcc-Ni with an increasing carbonization temperature and time. We note that stabilization of hcp-Ni by organic amines in a chemical reaction has been shown recently [17]. Graphitization of synthesized materials was observed by PXRD at and above 800 °C. Although the amount of graphite increased with the increasing temperature, it remained below that of nickel and the average crystallite size was small, below 20 nm.

Raman spectra (Figure 1b) confirmed the carbonization of the resin, as characteristic Raman bands of amorphous carbon appeared in the 1300–1600 cm^−1^ and 2500–3000 cm^−1^ region [18,19]. The broader band at 1332–1349 cm^−1^ and the narrower at 1592–1599 cm^−1^ were assigned to carbonic D and G bands, related to *sp*^3^-bonded carbon and graphitic *sp*^2^ carbon structures, respectively. The relatively low intensity of the G band indicated a low degree of graphitization, which was in agreement with PXRD measurements. The low intensity of second-order Raman bands in the 2500–3000 cm^−1^ region was also in agreement with this. Raman bands of amorphous carbon appeared on top of a broad luminescence band when carbonization was conducted at 500 °C, indicating an imperfect carbonization of the organic matrix at this temperature. This background, however, disappeared at higher temperatures. In general, the intensity of Raman bands decreased with increasing carbonization temperature, which indicated gradual replacement of *sp*^3^ carbon atoms with *sp*^2^ carbon in the carbonaceous support.

**Figure 2:**
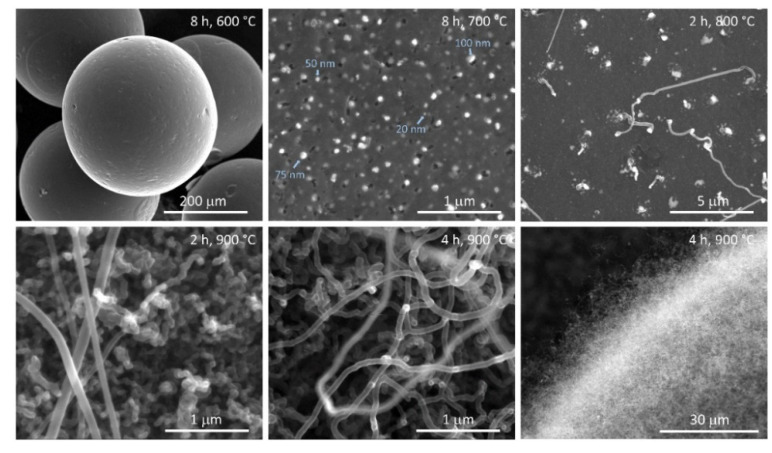
SEM images of Ni-NP/C microspheres (carbonization temperature and time are shown in images).

According to scanning electron microscopic (SEM) investigation, the synthesized Ni-NP/C catalysts inherited the spherical morphology of ion exchange resin, but shrunk from 600–900 µm (spheres of resin are not uniform) to 300–500 µm due to carbonization (Figure 2). Ni-NPs were clearly visible and were distributed all over the surface of microspheres. Carbon nanotube formation was detected at 800 °C carbonization, and it became very intensive at higher temperatures. The surface of microspheres was completely and densely covered by both curly and straight carbon nanotubes when carbonization was conducted at 900 or 1000 °C. Small nickel clusters are well-known to catalyze carbon nanotube formation, and this and the average crystallite size determined by PXRD suggest a wide crystallite size distribution of Ni-NPs in these microspheres.

The BET specific surface areas (SSA) of Ni-NP/C microspheres were determined by nitrogen adsorption measurements, and the results are shown in Table 1. Microspheres prepared by carbonizing the resin between 500 and 800 °C had small SSA, below 10 m^2^ g^−1^. SSA gradually increased with the increase in temperature to 900 and 1000 °C, which may be explained by the nanotube formation on microsphere surfaces.

### 2.2. Catalytic Activity of Ni-NP/C Microspheres for the Reduction of 4-Nitrophenol

**Figure 3:**
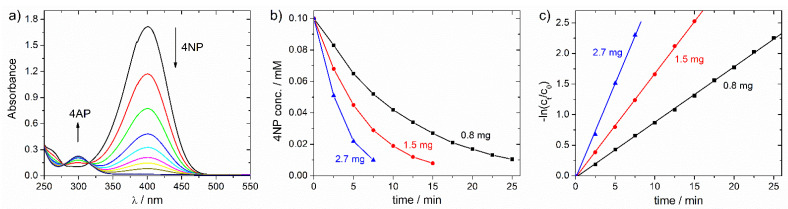
(**a**) Time dependent evolution of UV/vis spectra showing the decreasing amounts of 4NP anion in the Ni-NP/C catalyzed 4NP reduction reaction by NaBH_4_; (**b**) concentration of 4NP versus time and (**c**) −ln(c_t_/c_0_) versus time for the 4NP reduction reaction catalyzed by Ni-NP/C (amount of catalyst used is shown in Figures; initial 4NP and NaBH_4_ concentrations are 0.1 and 10 mM, respectively).

Ni-NP/C microspheres prepared by carbonizing the resin at 700 °C for 8 h were selected for testing catalytic activity, because Ni NPs were clearly visible on the microsphere surface by SEM (Figure 2) and nanotube formation was not observed at this temperature. This latter was expected to block the availability of free Ni surface for catalysis. The particle size of Ni NPs on microspheres’ surfaces was estimated by SEM to be between 10 and 110 nm, which was in agreement with the average particle size of 71 nm determined by PXRD (Table 1). The Ni content on microspheres’ surfaces was determined by dissolving surface Ni NPs in 1:1 diluted (34%) aqueous nitric acid and measuring the Ni ion content of the acidic solution by inductively coupled plasma optical emission spectroscopy (ICP-OES; surface Ni content was found to be 12 mg/g. The total Ni content of microspheres was determined by ICP-OES to be 118 mg/g, after digesting microspheres in hot chromic acid. 4NP was treated with NaBH_4_ in water without or with various amounts of catalyst at 20 °C; initial 4NP and NaBH_4_ concentrations were 0.1 mM and 10 mM, respectively. The reaction was monitored by UV/vis, following the decrease in the characteristic absorption band of nitrophenolate anion at 400 nm (Figure 3). No reaction was observed between 4NP and NaBH_4_ in the absence of Ni-NP/C. Similarly, no reaction was observed when Ni NPs were removed from the microsphere surfaces, by treatment with 1:1 diluted nitric acid and repeated washing, and the bare carbon microspheres were added to the reaction mixture. Upon addition of Ni-NP/C to the solution of 4NP and NaBH_4_, the absorption band of nitrophenolate anion decreased with time after an induction period. This induction period is well-known for this reaction, and related to the surface restructuring of the catalyst at the presence of 4NP [13,15,16]. To avoid this induction period [16], Ni-NP/C was added to the 4NP solution first, stirred for 10 min, and the NaBH_4_ solution was added consecutively. The 4NP reduction reaction was found to follow first-order kinetics, as indicated by the linear correlation of –ln(c_t_/c_0_) versus time, where c_t_ is the concentration of 4NP at time *t* and c_0_ is the 4NP initial concentration (Figure 3). The apparent rate constants were determined from the slope of fitted lines (*k*_app_ = 0.089, 0.166 and 0.307 min^−1^, using 0.8, 1.5 and 2.7 mg catalyst, respectively). The ratio of the apparent rate constant over the total weight of the Ni-NP/C catalyst (*m*_cat_) was found to be *k* = *k*_app_/*m*_cat_ = 111–113 min^−1^ g^−1^. The catalytic performances of various heterogeneous catalysts of the 4NP reduction reaction are compared in Table 2.

Ni-NP/C microspheres were found to be effective in catalyzing the 4NP reduction. After all of the 4NP was converted, Ni-NP/C spheres were fixed to the bottom of the reaction vessel using a permanent magnet, the solution was removed, and the reduction reaction was repeated using the same microspheres. No significant change in the apparent rate constant was observed during three consecutive cycles, which indicated a good reusability of the catalyst.

## 3. Materials and Methods

### 3.1. Materials

All starting materials and chemicals were used as received from commercial sources, and were of analytical or the highest purity reagent grade available (Sigma-Aldrich (Darmstadt, Germany), Fluka (Buchs, Switzerland), VWR (Darmstadt, Germany)). VARION BIM-7 commercial cation exchange resin was purchased from Iontech Kft Hungary (Litér, Hungary). The resin was styrene-based with 7% divinylbenzene crosslinker and 2% acrylonitrile modifier, and contained iminodiacetate (–CH_2_N(CH_2_COOH)_2_) functional groups. The resin had a nominal binding capacity of 1 mol divalent metal cation per 1 dm^3^ resin.

### 3.2. Synthesis of Ni-NP/C Microspheres

The commercial VARION BIM-7 resin was saturated with Ni^2+^ ions by performing consecutive conditioning (6 bed volume 1 M aq. NaOH solution), washing (1 bed volume distilled water), saturation (threefold excess of 1 M aq. NiSO_4_ solution), and final washing (water until washing liquid is Ni^2+^ free) steps [18]. The nickel ion saturated resin was dried in air, and then in a drying box at 120 °C for 24 h.

The dried and saturated resin (about 2 g in a porcelain combustion boat) was carbonized in a very slow inert nitrogen stream in a quartz tube, heated along 30 cm with an electric tube furnace. Nitrogen was taken from a commercial nitrogen cylinder (purity 99.996%) and passed through two consecutive columns to remove traces of oxygen (column packed with R3-11G BASF catalyst) and water (3A molecular sieves) before entering the quartz tube. The furnace was heated up to the desired temperature in 30 min, the temperature was kept constant for 2, 4, or 8 h, and then was left to cool down naturally. Synthesized products were used immediately or stored under nitrogen.

### 3.3. Catalyst Characterization

Powder X-ray diffraction (PXRD) measurements were performed on a Model PW 3710/PW 1050 Bragg-Brentano diffractometer (Philips, Eindhoven, The Netherlands) using Cu Kα radiation (λ = 1.541862 Å), secondary beam graphite monochromator, and proportional counter. Silicon powder (NIST SRM 640) and synthetic fluorophlogopite mica (NIST SRM 675) were used as internal standards. Lattice parameters were determined with Le Bail whole pattern decomposition method using the Full-prof Rietveld software suite.

Confocal Raman microscopic investigations were performed using a HORIBA JobinYvon LabRam HR instrument (Horiba, Kyoto, Japan). Raman spectra were recorded using He-Ne excitation (632 nm) and a laser power of 0.1 mW.

Scanning electron microscopy (SEM) was performed using a FEI Quanta 3D high-resolution microscope. The resolution was estimated to be <1.2 nm in high vacuum at 30 keV accelerating voltage. Energy dispersive X-ray analysis (EDX) was performed using the same instrument.

BET specific surface area (SSA) was determined using an RXM-100 Catalyst Characterization instrument (Advanced Scientific Design Inc. Bamco-Surplus, Texas City, TX, USA)) at liquid nitrogen temperatures, and using the volumetric method. Microspheres were pretreated before measurement at 300 °C for 2 h in vacuum.

UV/vis spectra were recorded on a Perkin-Elmer UV/vis Lambda 35 spectrometer (Perkin-Elmer GmbH, Überlingen, Germany) applying a slit wide of 1 nm.

The Ni^2+^ content of acidic aqueous solutions was determined by inductively coupled plasma optical emission spectrometry (ICP-OES) using a Spectro Genesis instrument (Spectro-Analytical Instruments GmbH, Kleve, Germany).

### 3.4. Reduction of 4-Nitrophenol (4NP)

A 0.2 mM aqueous 4NP and a 20 mM aqueous NaBH_4_ stock solution were prepared. The 4NP reduction was performed in a quartz cuvette with an optical path length of 1 cm. The cuvette was charged with the Ni-NP/C catalyst (0.8, 1.5, or 2.7 mg) and 1.5 cm^3^ 0.2 mM 4NP solution, stirred for 10 min, and then with 1.5 cm^3^ 20 mM NaBH_4_ solution. The reaction mixture was stirred and monitored by recording the UV/vis spectrum of the mixture from time to time (stirring was stopped during spectrum recording).

## 4. Conclusions

A simple and cost-effective pyrolytic method was developed in this work for the synthesis of carbon microspheres supported Ni nanoparticles (Ni-NP/C) for catalytic applications. The synthesis was based on carbonizing Ni-loaded, iminodiacetate functionalized, and styrene-based cation exchange resins. An advantage of the synthetic method is that spent ion exchange resins, or more favorably resins applied for nickel ion removal in water purification, could potentially be used as starting materials, thereby lowering the production cost. Ni-NP/C microspheres were characterized by various methods to obtain information about their phase composition and surface morphology. The selected Ni-NP/C microspheres, prepared by carbonizing the loaded resin at 700 °C, demonstrated excellent catalytic activity for the reduction of 4-nitrophenol. Due to the facile synthesis, the possibility to easily scale-up the production, and the reusability and separability of these catalysts, Ni-NP/C microspheres are expected to replace more expensive catalysts in future catalytic applications.

## Figures and Tables

**Table 1:** Crystalline phase composition relative to the total nickel content of the carbonized resin.

Carbonization		hcp-Ni^1^	fcc-Ni^1^	Graphite ^1^	SSA ^2^
°C	Hours	at% (nm)	at% (nm)	at% (nm)	m^2^ g^−1^
500	2 4 8	55 (10) 50 (10) 50 (10)	45 (8) 50 (8) 50 (8)		3 5 8
600	2 4 8	40 (14) 30 (11) 30 (11)	60 (11) 70 (14) 70 (17)		5 7 8
700	2 4 8	15 (11) 8 (11) 5 (11)	85 (47) 92 (47) 95 (71)		4 7 9
800	2 4 8		100 (66) 100 (66) 100 (99)	14 (12) 14 (12) 18 (17)	7 4 6
900	2 4 8		100 (82) 100 (99) 100 (99)	25 (12) 25 (12) 33 (17)	15 15 29
1000	2 4 8		100 (71) 100 (123) 100 (123)	67 (12) 67 (12) 82 (14)	39 57 55

^1^ Relative to the total Ni content, in at%. Average crystallite size in nm is provided in parenthesis; estimated using the Scherrer equation. ^2^ Specific surface area.

**Table 2:** Comparison of reaction conditions and rate constants, *k* = *k*_app_/*m*_cat_, for the reduction of 4NP with NaBH_4_ at the presence of supported, composite or bare Ni NPs ^1^.

Catalyst	4NP Conc. mM	NaBH_4_ Conc. mM	Cat. Amount mg cm^−3^	Temp. °C	Rate Constant ^1^ min^−1^ g^−1^	Reference
Ni-NP	0.1	30	0.065	r.t.	1.3	[20]
Ni-NP/RGO	0.1	30	0.1	r.t.	1.5	[20]
Ni-NP/hydrogel	2.16	216	0.48	27	0.9	[21]
Ni-NP/hydrogel	14	288	1	30	1.1	[22]
Ni-NP/polymer-sponge	0.13	200	6.7	r.t.	7.9	[23]
Ni-NP	0.1	30	0.01	r.t.	1.8	[24]
Ni-NP/RGO	0.1	30	0.01	r.t.	6.6	[24]
Ni-NP	0.05	20	0.005	r.t.	9.4	[25]
Ni-NP/RGO	0.05	20	0.005	r.t.	25	[25]
Ni-NP/CTAB + gelatin	0.09	18	0.9	20	48	[26]
Ni-NP/CTAB + PEG10000	0.09	18	0.9	20	54	[26]
Ni-NP/silica	0.09	18	0.9	20	20–56	[27]
Ni-NP/silica	0.145	7.5	0.1	20	13–297	[28]
Ni-NP/carbon black	0.5	53	0.02	30	14–597	[29]
Ni-NP/carbon spheres	0.1	10	0.3–0.9	20	111–113	this work

^1^ Calculated from data provided in original references. The total mass of supported/composite Ni NP catalysts is shown (Ni content can be found in referenced papers). Abbreviations: r.t. = room temperature, RGO = reduced graphene oxide, CTAB = cetyltrimethyl ammonium bromide, PEG = polyethylene glycol.

## Data Availability

The data presented in this study are available from the authors.
